# Concentrations of macronutrients, minerals and heavy metals in home-prepared diets for adult dogs and cats

**DOI:** 10.1038/s41598-019-49087-z

**Published:** 2019-09-10

**Authors:** Vivian Pedrinelli, Rafael Vessecchi Amorim Zafalon, Roberta Bueno Ayres Rodrigues, Mariana Pamplona Perini, Renata Maria Consentino Conti, Thiago Henrique Annibale Vendramini, Júlio César de Carvalho Balieiro, Márcio Antonio Brunetto

**Affiliations:** 1 School of Veterinary Medicine and Animal Science, University of Sao Paulo (USP) – Department of Medical Clinic, São Paulo, postcode 13690-970 Brazil; 2 School of Veterinary Medicine and Animal Science, University of Sao Paulo (USP) – Department of Animal Nutrition and Production, Pirassununga, postcode 13635-900 Brazil

**Keywords:** Calcium, Potassium

## Abstract

Pet owners often don’t acknowledge the need for home-prepared diet formulation by a trained professional and may use recipes from sources such as the internet. Macronutrient and mineral composition of home-prepared diets were analyzed and compared to NRC and FEDIAF recommendations, and heavy metal concentrations were analyzed and compared to FDA maximum tolerable levels (MTL) for dogs and cats. Recipes of home-prepared diets for adult dogs (n = 75) and cats (n = 25) were evaluated. Analyses of protein, fat, and fiber were performed according to AOAC, and mineral and heavy metal analyses were performed using inductively coupled plasma optical emission spectrometry (ICP-OES). None of the diets supplied recommended levels of all nutrients evaluated, and more than 84.0% of diets presented three or more nutrients below recommendations. Nutrients with most levels below recommendations were calcium and potassium in recipes for dogs and iron and zinc in recipes for cats. As for heavy metals, levels of lead, cobalt, mercury, uranium, and vanadium were above MTLs. Results suggest that home-prepared diets may be a health risk to dogs and cats if not properly formulated. Furthermore, the chronic heavy metal intake must be better elucidated in order to understand the full impact of results.

## Introduction

A diet that is nutritionally balanced is essential for the maintenance of animal health and increasing longevity. The National Research Council^1^ and European Pet Food Industry^2^ are two of the main publications for the nutritional requirements of dogs and cats. To ensure optimal nutrition, however, there are other factors related to the diet that must be considered, which include ingredient safety, preparation, and storage^3,4^. Nutrient requirements are established by gathering evidence of deficiency and toxicity at certain levels, and the values in between the minimum and maximum recommended levels represent a safe and adequate range of nutrient intake, which may vary according to the nutrient in question and diet composition^1,2,5^.

For humans, the choice of food to be consumed is a complex act that involves social and cultural aspects, which can also influence food choices for dogs and cats^6^. A study conducted by Laflamme *et al*.^7^ observed that 18% of animals that took part in a survey in the United States and Australia were fed home-prepared diets as a part of the diet or exclusively. A more recent study^8^ applied a questionnaire to more than 3.000 owners of dogs and cats from 55 countries and observed that more than 60% fed their pets home-prepared food as a part of the diet, and 12% of dog owners ant 6% of cat owners fed exclusively home-prepared diets.

The main reasons that may motive owners to change to a home-prepared diet are diseases that require dietary change, difficulty to interpret labels, concern about preservatives and better palatability^6,9–11^. But sometimes owners are not aware that homemade diets need complex preparation, specific ingredients, and supplements and may be more expensive than extruded pet food^12^. One study^13^ evaluated the cost of extruded diets, wet diets and home-prepared diets for adult dogs in the United States, and concluded that an extruded diet is the least expensive type of food, followed by home-prepared diet. However, when considering canine therapeutic renal diets^14^, home-prepared was less expensive than both extruded and wet diets.

The first step to evaluate the adequacy of a homemade diet is a thorough dietary history. This can indicate manufacturer, brand, amount of each ingredient offered, preparation mode, storage and number of daily meals^3,11^. When evaluating homemade diets, the main concerns are if the nutritional profile of the diet is adequate, if the owner is preparing the recipe according to instructions and if there was a change to the original recipe^11,12^. Checking for nutritional adequacy of a diet for dogs and cats can be challenging for professionals of veterinary medicine and animal science. There are three methods: diet formulation on a computer software based on nutrient requirements; bromatological and laboratory analysis of the diet; and *in vivo* studies^1,15^. On the contrary of what some might presume, few deficiencies can be detected with routine exams like complete blood count, biochemistry and urine exam^12^.

The risk of developing diseases related to malnutrition exists if a dog or cat consumes an imbalanced diet, regardless of the manufacturer, how the product is marketed or even if the professional has previously heard of the product^11^. Few studies evaluated the nutritional adequacy of home-prepared diets. Streiff *et al*.^16^ performed a laboratory analysis of home-prepared diets for puppies and adult dogs from Vienna, Austria, and diets for both age ranges presented calcium, copper, phosphorus, potassium, zinc and vitamin E below AAFCO recommendations. Another study^17^ conducted in the United States evaluated 200 recipes of homemade diets published in websites and books in a diet formulation software (Balance IT Autobalancer, California, USA). It was observed that 95% of the recipes had at least one nutrient that did not meet requirements and 83.5% presented multiple nutrients below NRC recommendations. Similar results were found in a study conducted in Brazil^18^, in which none of the more than 100 recipes supplied all nutrients according to FEDIAF recommendations. Homemade diets intended for dogs with cancer were also evaluated in computer software^19^, and observed that none of the 27 diets used in the study met all NRC recommendations. The nutritional composition of diets for dogs and cats with chronic kidney disease was evaluated by Larsen *et al*.^20^. As expected, recipes presented lower levels of protein. However, more than half of the diets evaluated did not meet the requirements for choline, calcium, zinc, and cobalamin.

The consequences of nutritional imbalances are diverse. One of the most common nutritional diseases is the nutritional secondary hyperparathyroidism (NSHP) due to calcium deficiency and calcium:phosphorus ratio of less than 1:1. Clinical signs of this disease include micro and complete bone fractures and may take months in young animals and up to years in adults^21–23^. Other deficiencies, such as zinc, phosphorus, and potassium have also been reported^24–28^.

Another important point to consider when considering food safety is the contamination of heavy metals, that by definition are metals and semimetals that present potential toxic effects for humans, animals and the environment^29,30^. The Agency for Toxic Substances and Disease Registry, from the United States, considers as potentially toxic 20 elements, including aluminum, arsenic, boron, barium, cobalt, lead, mercury, uranium, and vanadium^31^. The concentration of heavy metals in food depends on several factors, such as the condition of the environment in which the food was cultivated, soil composition, exposure to fertilizers, and processing^32^. Most heavy metals are absorbed by inhalation or via the gastrointestinal tract, and some may bioaccumulate in plasma proteins, liver, kidneys, bones, hair, and adipose tissue^30,33^. The toxicity of a metal is related to its accumulation in tissues and depends on the frequency of exposure, the amount absorbed and channel of absorption. Other factors that interfere in the toxicity of heavy metals are the chemical form, which may interfere in bioavailability; the age of the individual; and interaction between elements and nutrients and other metals^30,33,34^.

Few studies evaluated concentrations of heavy metals in pet food. Fernandes *et al*.^35^ evaluated 95 extruded diets for dogs and puppies in Brazil and observed that aluminum, antimony, and uranium were present in some samples above the human maximum permitted levels. The authors also observed that all samples that presented high antimony concentrations contained food coloring. The United States Food and Drug Administration (FDA) published in 2011 a review of heavy metal safety for food intended for dogs and cats^36^, in which maximum tolerable levels (MTL) are presented for several heavy metals for these species. MTL is defined as the amount of the metal that, when ingested by a period of time, will not impair the health or performance of animals^37^. However, few studies were made regarding the consequences of chronic heavy metal intake in dogs and cats.

The present study aimed to evaluate macronutrient and mineral composition of recipes of home-prepared diets for dogs and cats published on the internet and evaluate their nutritional adequacy in comparison to NRC^1^ and FEDIAF^2^ recommendations for healthy adults.

## Methods

### Selection of recipes

Recipes for healthy adult dogs and cats published in Portuguese were selected using Google browser, and search terms were “home-prepared diet”, “home-cooked diet”, “homemade food”, “home-prepared diet recipe”, “home-cooked diet recipe” and “homemade food recipe”, all followed by the terms “dog” and “cat”. Only recipes until the 10^th^ page of the browser for each term were considered.

As exclusion criteria for this study, recipes were not considered if not intended for healthy adults, if it was stated that they were not meant for daily use, if considered by recipe’s author as a snack or milk replacer, and if the quantity of one or more ingredients was not specified. Recipes remaining after applying exclusion criteria were numbered for each species and then 75 recipes for dogs and 25 recipes for cats were drafted to be evaluated.

### Preparation of selected recipes

Ingredients were acquired from three different markets in the city of Sao Paulo, Brazil, and preference was given to fresh foods. When fresh food was not available, frozen foods were acquired. Preparation of 500 grams samples for recipes was done according to recipe’s instruction of ingredients, amounts and cooking mode.

All ingredients were weighed on a digital scale and then blended with the use of a food processor. When recipes indicated units of an ingredient, USDA^38^ measures were used. If supplementation was indicated but there was no specification of manufacturer, brand or amount, a commercial powdered supplement for homemade diets (Food Dog Adulto Manutenção, Botupharma, Botucatu, SP, Brazil) was used, and manufacturer’s recommended amount was included.

### Bromatological analyses

Samples were dehydrated in forced circulation oven at 55 °C for 72 hours^39,40^. Afterward, they were ground and put in forced circulation oven at 105 °C to determine dry matter content. Crude protein analyses were performed by the Kjeldahl method, crude fat was determined by the Soxhlet method and ash content was determined by incineration at 550 °C^30,31^. Crude fiber was determined by Weende method^41^. Nitrogen-free extract (NFE) was calculated by subtracting ash, crude fiber, crude protein, and crude fat percentages out of 100 grams of dry matter^1^. All analyses were performed in duplicate at the Multiuser Laboratory of Animal Nutrition and Bromatology of the Department of Nutrition and Animal Production of the School of Veterinary Medicine and Animal Science of University of Sao Paulo, Pirassununga - Brazil.

### Mineral and heavy metal analyses

For mineral analyses, 200 mg of samples were put in 100 mL assay tubes, and 4 mL of nitric acid (HNO_3_) was added and then tubes were left to rest for 30 minutes. After this period, samples were heated in hot plates until the reduction of half of the volume. Hot plates were then turned off and, after cooling, 1 mL of perchloric acid (HClO_4_) was added to each tube. Samples were reheated until a sample size of 2 mL was reached^42^. This digestion method was performed at the Multiuser Laboratory of Animal Nutrition and Bromatology of the Department of Nutrition and Animal Production of the School of Veterinary Medicine and Animal Science of the University of Sao Paulo.

Close vessel microwave digestion was used to process samples for heavy metal analyses. Samples of 0.5 g of recipes were put in polypropylene tubes, and 1.5 mL of HNO_3_ and 2 mL of hydrogen peroxide (H_2_O_2_) were added to each sample. Tubes rested for 30 minutes and then 4.5 mL of ultrapure water was added. Tubes were then put in the microwave (Multiwave GO, Anton Parr, Graz, Austria) and were heated in two phases: the first phase, samples were heated for 20 minutes until 180 °C with radiofrequency power of 400 W; the second phase, samples were heated for 10 minutes at 180 °C with radiofrequency power of 800 W. After this period, samples were left to cool for 10 minutes. This digestion method was performed at the laboratory of Biorigin Brazil (Lençóis Paulista, Brazil).

Analyses of minerals were performed in triplicate by inductively coupled plasma optical emission spectrometry [ICP-OES (ICPE-9000, Shimadzu of Brazil, Barueri, SP, Brazil)] at the Multiuser Laboratory of Animal Nutrition and Bromatology of the Department of Nutrition and Animal Production of the School of Veterinary Medicine and Animal Science of University of Sao Paulo (Pirassununga, Brazil). Operational conditions are presented in Table 1. For the determination of antimony, arsenic, selenium, and mercury a hydride generator (hydrideI CP, Elemental Scientific, Omaha, NE, United States) was coupled to the ICP-OES.

**Table 1:** Operational conditions of inductively coupled plasma optical emission spectrometry (ICP-OES) with axial configuration.

Parameter	Characteristics
Radiofrequency power (W)	1200
Plasma gas flow rate (L/min)	10
Auxiliary gas flow rate (L/min)	0.6
Sample uptake rate (s)	30
Nebulizer gas flow rate (L/min)	0.7
Nebulizer type	Concentric
Spray chamber	Cyclone
Replicates	3

Preparation of external calibration curves was done by using multielement standard solutions at concentrations of 100 mg/L for arsenic (As), aluminum (Al), boron (B), barium (Ba), beryllium (Be), calcium (Ca), cadmium (Cd), cobalt (Co), chromium (Cr), copper (Cu), iron (Fe), mercury (Hg), potassium (K), magnesium (Mg), manganese (Mn), selenium (Se), sodium (Na), nickel (Ni), phosphorus (P), lead (Pb), antimony (Sb), tin (Sn), vanadium (V) and zinc (Zn) (SpecSol, Quimilab, Jacareí, SP, Brazil) and single element solutions of 100 mg/L for uranium (U). Curves were prepared in a range of concentrations from 0.1 to 5 mg/L for Cu, Zn, Na and Mn, from 0.5 to 100 mg/L for Ca, P, Mg and K and from 0.001 to 2 mg/L for arsenic, Al, B, Ba, Be, Cd, Co, Cr, Fe, Hg, Ni, Pb, Sb, Se, Sn, U and V.

Analyses of chloride and iodine were not performed. The methodology used in the present study does not allow chloride and iodine evaluation due to high ionization energy necessary, as less than 30% of atoms of both these elements are ionized in argonium plasma^43^.

### Statistical analyses

Descriptive data were calculated as frequencies (%), and the Shapiro-Wilk test was used to test the normality of variables. For normally distributed variables Student’s t-test was used to evaluate the significant effect between analyses results and NRC^1^ and FEDIAF^2^ nutritional recommendations for protein, fat, and minerals. For variables with non-normal distribution, the Wilcoxon test was used for the same comparisons. NRC^1^ and FEDIAF^2^ recommendations for inactive adult dogs and cats for 1000 kcal were used, and daily energy intake of 95 kcal/kg^0.75^ for dogs and 75 kcal/kg^0.67^ for cats were considered. Statistical significance was accepted if *P* ≤ 0.05.

For analyses of heavy metals, maximum tolerable levels (MTL) recommended by FDA^36^ were considered. FDA does not present a recommendation of NMT for Al, B, Ba, and Sn, therefore values corresponding to the most sensitive mammal according to NRC^37^ were considered for comparison. Coefficients of correlation between ingredient inclusion in dry matter basis and heavy metal concentration were performed by Pearson correlation, which was considered low if coefficients were <0.5, moderate if coefficients were between 0.5 and 0.7 and high if coefficients were >0.7. Data were analyzed using SAS version 9.4 (SAS Institute, NC, USA).

## Results

### Recipes selected

A total of 100 recipes of 35 different sources were included in this study, 75 for dogs and 25 for cats. Of recipes intended for dogs, 12.0% (n = 9/75) did not contain meat and were considered vegetarian, and 6.6% (n = 5/75) did not contain any animal products and were considered vegan. Of recipes intended for cats, 4.0% (n = 1/25) were vegetarian and 8.0% (n = 2/25) were vegan. As for cooking, 2.7% (n = 2/75) of recipes for dogs and 20.0% (n = 5/25) for cats contained raw animal products, and the remaining diets had cooked animal products. Only 20.0% (n = 15/75) of recipes for dogs indicated vitamin-mineral supplement as an ingredient, of which 18.67% (n = 14/75) did not specify manufacturer or product, and 53.3% (n = 40/75) of recipes for dogs did not indicate any supplementation of minerals, amino acids or vitamins. None of the recipes for cats indicated vitamin-mineral supplementation and 32.0% (n = 8/25) did not indicate any supplementation of minerals, amino acids or vitamins.

The ingredients used most often in recipes for dogs were: carrot (n = 35/75; 46.7%); white rice (n = 24/75; 32.0%); whole egg (n = 20/75; 26.7%); skinless chicken breast (n = 19/75; 25.3%); and zucchini (n = 17/75; 22.7%). In recipes for cats, the ingredients most often used were: whole egg (n = 10/25; 40.0%); beef heart (n = 10/25; 40.0%); carrot (n = 8/25; 32.0%); skinless chicken breast (n = 8/25; 32.0%); and bovine liver (n = 7/25; 28.0%).

### Nutritional analyses

None of the diets for either dogs or cats met all requirements for protein, fat, and minerals recommended by NRC^1^ and FEDIAF^2^. When compared to NRC^1^, 84.0% (n = 63/75) of recipes for dogs and all of the recipes for cat presented three or more nutrients below recommended levels. When compared to FEDIAF^2^, all of the recipes for dogs and cats presented three or more nutrients below recommendations.

**Figure 1:**
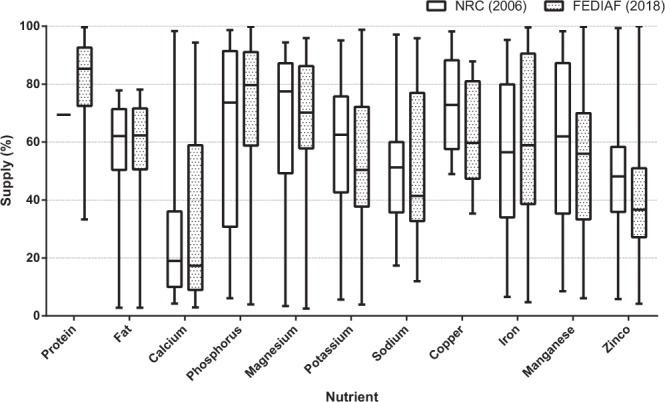
Box-and-whisker plot of percentage of nutrient supply of recipes for dogs with nutrient levels below NRC^1^ and FEDIAF^2^ recommendations. Boxes represent interquartile range from 25^th^ to 75^th^ percentile, horizontal lines within boxes represent median, and bars above and below boxes represent maximum and minimum value points, respectively.

**Figure 2:**
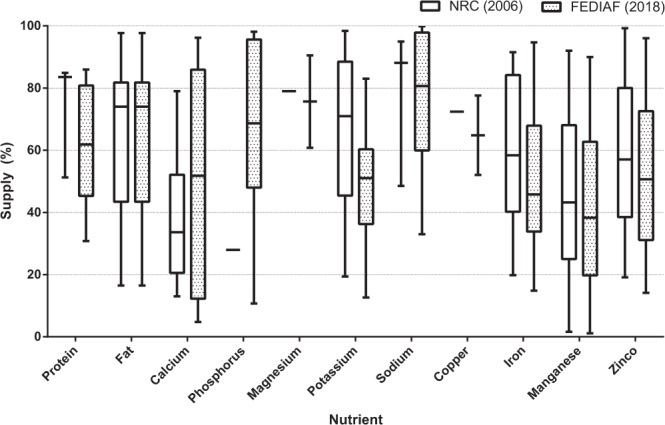
Box-and-whisker plot of percentage of nutrient supply of recipes for cats with nutrient levels below NRC^1^ and FEDIAF^2^ recommendations. Boxes represent interquartile range from 25^th^ to 75^th^ percentile, horizontal lines within boxes represent median, and bars above and below boxes represent maximum and minimum value points, respectively.

Results for macronutrient and mineral analyses of recipes for dogs compared to minimum recommendations are listed in Table 2, and the results of recipes for cats are listed in Table 3. The complete list of results for all diets can be found in Supplementary Tables S1 and S2. As for NRC^1^ maximum levels, none of the recipes for dogs and one recipe for cats (4.0%) had levels of fat above 82.5 g/1000 kcal. When compared to FEDIAF^2^ maximum recommended levels, one recipe for dogs (1.3%) had levels of calcium above 6.25 g/1000 kcal; none of the recipes for dogs presented phosphorus levels above 4.0 g/1000 kcal; and 11 recipes for dogs (14.7%) and 5 recipes for cats (20.0%) had calcium:phosphorus ratio above 2:1. The supply of nutrients was also evaluated for recipes for dogs (Fig. 1) and for cats (Fig. 2) that were below recommendations.

**Table 2:** Results of macronutrient and mineral analyses (in 1000 kcal) of 75 home-prepared recipes for healthy adult dogs in comparison to NRC^1^ and FEDIAF^2^ recommendations for adults.

	NRC^1^	FEDIAF^2^	Mean	SD	Range	*P* NRC	*P* FEDIAF	% below minimum (n)	
	Minimum	Minimum						NRC	FEDIAF
DM (g/100 g)	—	—	33.52	15.00	15.65–91.05	—	—	—	—
Protein (g)	25.00	52.10	87.47	32.33	17.36–157.01	<0.0001	<0.0001	1.33 (1)	18.67 (14)
Fat (g)	13.80	13.75	28.18	16.29	0.38–79.32	<0.0001	<0.0001	16.00 (12)	16.00 (12)
Crude fiber (g)	—	—	10.20	8.61	0.68–52.06	—	—	—	—
Ash (g)	—	—	10.46	9.20	1.91–80.28	—	—	—	—
NFE (g)	—	—	99.13	48.22	0.53–231.76	—	—	—	—
Calcium (g)	1.00	1.45	0.91	1.24	0.04–6.86	0.0054	<0.0001	69.33 (52)	82.67 (62)
Phosphorus (g)	0.75	1.16	1.13	0.44	0.04–2.39	<0.0001	<0.0001	17.33 (13)	53.33 (40)
Ca:P ratio	—	½	0.88	1.22	0.04–5.85	—	0.0018	—	76.00 (57)
Potassium (g)	1.00	1.45	0.80	0.39	0.05–1.81	0.0056	<0.0001	68.00 (51)	94.67 (71)
Magnesium (g)	0.15	0.20	0.22	0.11	0.01–0.48	<0.0001	<0.0001	26.67 (20)	50.67 (38)
Sodium (g)	0.20	0.29	0.42	0.40	0.03–1.81	<0.0001	0.3480	36.00 (27)	54.67 (41)
Copper (mg)	1.50	2.08	9.08	9.57	0.73–64.51	<0.0001	<0.0001	13.33 (10)	18.67 (14)
Iron (mg)	7.50	10.40	11.96	9.27	0.49–48.23	0.0001	0.9666	33.33 (25)	56.00 (42)
Manganese (mg)	1.20	1.67	3.53	3.38	0.10–17.10	<0.0001	0.0002	32.00 (24)	40.00 (30)
Zinc (mg)	15.00	20.80	12.72	9.07	0.88–47.13	0.0127	<0.0001	66.67 (50)	78.67 (59)

SD. standard deviation; NFE. nitrogen-free extract.

**Table 3:** Results of macronutrient and mineral analyses (in 1000 kcal) of 25 home-prepared recipes for healthy adult cats in comparison to NRC^1^ and FEDIAF^2^ recommendations for adults.

	NRC^1^	FEDIAF^2^	Mean	SD	Range	*P* NRC	*P* FEDIAF	% below minimum (n)	
	Minimum	Minimum						NRC	FEDIAF
DM (g/100 g)	—	—	26.57	7.62	6.20–39.13	—	—	—	—
Protein (g)	50.00	83.30	124.58	54.78	25.64–210.52	<0.0001	0.0009	12.00 (3)	24.00 (6)
Fat (g)	22.50	22.50	33.99	19.36	3.72–87.88	0.0067	0.0067	32.00 (8)	32.00 (8)
Crude fiber (g)	—	—	9.92	9.20	1.66–37.88	—	—	—	—
Ash (g)	—	—	13.07	5.46	5.29–29.75	—	—	—	—
NFE (g)	—	—	53.19	65.02	1.07–216.46	—	—	—	—
Calcium (g)	0.72	1.97	1.63	1.32	0.09–5.36	0.0021	0.2171	32.00 (8)	68.00 (17)
Phosphorus (g)	0.64	1.67	1.31	0.47	0.18–2.10	<0.0001	0.0010	4.00 (1)	76.00 (19)
Ca:P ratio	—	½	1.55	1.95	0.06–9.51	—	0.5209	—	40.00 (10)
Potassium (g)	1.30	2.00	1.04	0.44	0.25–2.13	0.0070	<0.0001	80.00 (20)	96.00 (24)
Magnesium (g)	0.10	0.13	0.24	0.09	0.08–0.52	<0.0001	<0.0001	4.00 (1)	8.00 (2)
Sodium (g)	0.17	0.25	0.65	0.43	0.08–1.50	<0.0001	0.0001	12.00 (3)	28.00 (7)
Copper (mg)	1.20	1.67	12.21	12.21	0.87–52.26	0.0001	0.0002	4.00 (1)	8.00 (2)
Iron (mg)	20.00	26.70	13.38	5.60	3.96–25.29	<0.0001	<0.0001	88.00 (22)	100.00 (25)
Manganese (mg)	1.20	1.67	2.44	4.17	0.02–15.88	0.6472	0.1884	64.00 (16)	76.00 (19)
Zinc (mg)	18.50	25.00	15.81	8.71	3.54–35.28	0.1356	<0.0001	68.00 (17)	84.00 (21)

SD. standard deviation; NFE. nitrogen-free extract.

Selenium was below detection levels in 98 of all the 100 recipes included and therefore was not used for comparison.

### Heavy metal analyses

ICP-OES methodology allowed the evaluation of 15 different heavy metals in recipes for dogs and cats. Antimony and tin were detected in all diets for dogs, and boron was not observed above the detection limit of 0.001 mg/kg in any recipe (Table 4). Aluminum, chromium, mercury, lead, antimony, tin and uranium were detected in all recipes for cats, beryllium was detected in only one diet and boron was not observed above the detection limit of 0.001 mg/kg in any recipe (Table 5).

**Table 4:** Heavy metal concentration in recipes for dogs and comparison to maximum tolerable levels (MTL).

	Heavy metal concentration (mg/kg of DM)				
	MTL	Mean ± SD	Range	% above MTL (n)	% samples with detection (n)
Aluminum (Al)	200^a^	26.04 ± 25.37	<0.001–159.48	0.0 (0)	97.3 (73)
Arsenic (As)	12.5^b^	0.17 ± 0.09	<0.001–0.58	0.0 (0)	18.7 (14)
Boron (B)	150^a^	—	—	0.0 (0)	0.0 (0)
Barium (Ba)	100^a^	4.93 ± 3.80	<0.001–15.02	0.0 (0)	97.3 (73)
Beryllium (Be)	5^b^	0.43 ± 0.19	<0.001–1.52	0.0 (0)	8.0 (6)
Cadmium (Cd)	10^b^	0.61 ± 0.57	<0.001–2.62	0.0 (0)	84.0 (63)
Cobalt (Co)	2.5^b^	1.06 ± 0.68	<0.001–3.08	4.0 (3)	89.3 (67)
Chromium (Cr)	10^b^	2.15 ± 1.11	<0.001–6.58	0.0 (0)	97.3 (73)
Mercury (Hg)	0.27^b^	0.76 ± 1.07	<0.001–6.07	70.7 (53)	92.0 (69)
Nickel (Ni)	50^b^	1.53 ± 2.27	<0.001–19.44	0.0 (0)	86.7 (65)
Lead (Pb)	10^b^	8.28 ± 4.67	<0.001–20.07	26.7 (20)	89.3 (67)
Antimony (Sb)	40^b^	1.13 ± 0.59	0.13–3.27	0.0 (0)	100.0 (75)
Tin (Sn)	100^a^	6.94 ± 2.71	0.53–16.42	0.0 (0)	100.0 (75)
Uranium (U)	10^b^	68.49 ± 40.57	<0.001–173.36	92.0 (69)	97.3 (73)
Vanadium (V)	1^b^	1.51 ± 1.12	<0.001–5.02	53.3 (40)	84.0 (63)

MTL. maximum tolerable level; SD. standard deviation; ^a^reference level for most sensitive mammal according to *National Research Council*^37^; ^b^reference level for most sensitive mammal according to *United States Food and Drug Administration*^36^.

**Table 5:** Heavy metal concentration in recipes for cats and comparison to maximum tolerable levels (MTL).

	Heavy metal concentration (mg/kg of DM)				
	MTL	Mean ± SD	Range	% above MTL (n)	% samples with detection (n)
Aluminum (Al)	200^a^	24.46 ± 21.97	2.11–90.49	0 (0.0)	100.0 (25)
Arsenic (As)	12,5^b^	0.27 ± 0.09	<0.001–0.36	0 (0.0)	12.0 (3)
Boron (B)	150^a^	—	—	0 (0.0)	0.0 (0)
Barium (Ba)	100^a^	3.67 ± 3.61	<0.001–14.83	0 (0.0)	92.0 (23)
Beryllium (Be)	5^b^	0.13 ± 0.00	<0.001–0.13	0 (0.0)	4.0 (1)
Cadmium (Cd)	10^b^	1.22 ± 1.03	<0.001–3.31	0 (0.0)	92.0 (23)
Cobalt (Co)	2,5^b^	1.00 ± 0.61	<0.001–2.60	4.0 (1)	96.0 (24)
Chromium (Cr)	10^b^	2.61 ± 1.18	0.73–6.44	0 (0.0)	100.0 (25)
Mercury (Hg)	0,27^b^	0.78 ± 0.76	0.07–3.66	76.0 (19)	100.0 (25)
Nickel (Ni)	50^b^	1.66 ± 2.20	<0.001–12.00	0 (0.0)	96.0 (24)
Lead (Pb)	10^b^	10.37 ± 5.25	1.26–24.52	44.0 (11)	100.0 (25)
Antimony (Sb)	40^b^	1.29 ± 0.49	0.61–2.16	0 (0.0)	100.0 (25)
Tin (Sn)	100^a^	8.60 ± 2.83	3.45–13.35	0 (0.0)	100.0 (25)
Uranium (U)	10^b^	81.17 ± 44.83	11.68–169.01	100.0 (25)	100.0 (25)
Vanadium (V)	1^b^	1.87 ± 1.36	<0.001–4.56	60.0 (15)	88.0 (22)

MTL. maximum tolerable level; SD. standard deviation; ^a^reference level for most sensitive mammal according to *National Research Council*^37^; ^b^reference level for most sensitive mammal according to *United States Food and Drug Administration*^36^.

**Figure 3:**
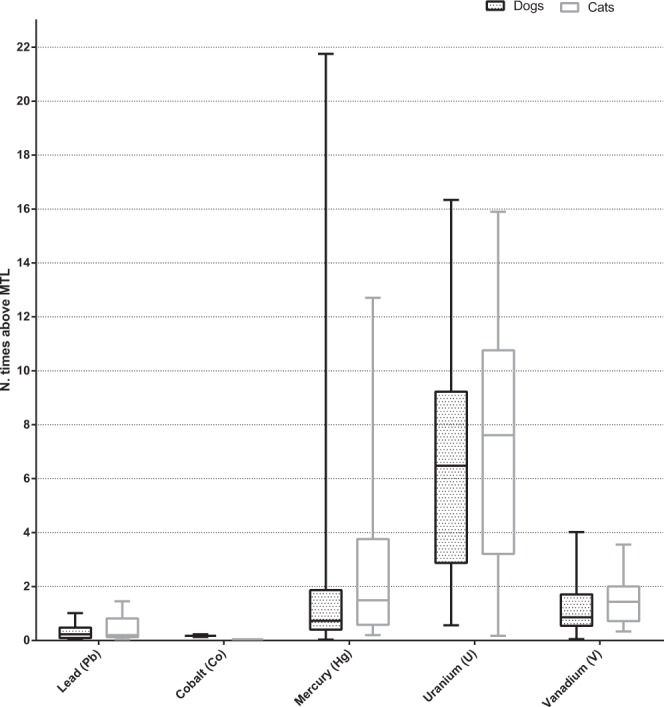
Box-and-whisker plot of percentage of nutrient supply of recipes for dogs and cats with heavy metal concentrations above maximum tolerable levels (MTL)^36,37^. Boxes represent interquartile range from 25^th^ to 75^th^ percentile, horizontal lines within boxes represent median, and bars above and below boxes represent maximum and minimum value points, respectively.

Lead, cobalt, mercury, uranium, and vanadium were the only heavy metals with concentrations above MTL in recipes for both dogs and cats (Fig. 3). In diets for dogs, soy flour and spinach were positively correlated to these five heavy metal concentrations, and barley was positively correlated to cobalt, lead, uranium and vanadium concentrations. Inclusion of olive oil and bean were highly correlated to mercury concentrations (r^2^ = 0.96; p = 0.001 and r^2^ = 0.87; p = 0.025, respectively), and the vitamin-mineral supplement was moderately correlated to cobalt concentrations (r^2^ = 0.65; p = 0.001). In diets for cats, beetroot, bell pepper, collards, and yam were positively correlated to all five heavy metals above MTLs. Beef liver and chicken breast meat were highly correlated to cobalt concentrations (r^2^ = 0.80; p = 0.030 and r^2^ = 0.81; p = 0.050, respectively). Beef liver, carrot and chicken breast meat were highly correlated to lead concentrations (r^2^ = 0.79; p = 0.035, r^2^ = 0.71; p = 0.048 and r^2^ = 0.86; p = 0.030, respectively). As for uranium concentrations, inclusion of carrot, celery, and chicken breast meat were highly correlated (r^2^ = 0.72; p = 0.045, r^2^ = 1.00; p = 0.027, and r^2^ = 0.84; p = 0.037, respectively). Carrot and celery inclusions were highly correlated to vanadium (r^2^ = 0.71; p = 0.048 and r^2^ = 1.00; p = 0.019, respectively). All moderate and high correlations between ingredients and concentrations of these heavy metals can be found in Tables 6 and 7.

**Table 6:** Correlation between ingredient inclusion in dry matter basis and heavy metal concentration in diets for dogs, for ingredients with one or more moderately or highly positive correlation (r^2^ ≥ 0.5).

	Heavy metal									
	Cobalt (Co)		Mercury (Hg)		Lead (Pb)		Uranium (U)		Vanadium (V)	
	r^2^	p	r^2^	p	r^2^	p	r^2^	p	r^2^	p
Barley (n = 4)	0.60	0.396	−0.24	0.762	0.68	0.322	0.80	0.198	0.87	0.128
Beef heart (n = 4)	−0.80	0.203	−0.15	0.849	−0.73	0.274	−0.90	0.095	−0.91	0.087
Beetroot (n = 3)	−0.82	0.388	0.83	0.375	−0.44	0.713	−0.97	0.154	0.86	0.343
Broccoli (n = 7)	0.32	0.479	0.53	0.224	0.13	0.780	0.16	0.731	0.11	0.820
Lentil (n = 3)	0.98	0.122	−0.60	0.588	−0.44	0.711	−0.13	0.914	−0.15	0.901
Olive oil (n = 8)	0.21	0.610	0.96	0.000	0.02	0.956	0.01	0.989	−0.03	0.937
Pea (n = 4)	−0.66	0.341	0.55	0.452	−0.57	0.430	−0.54	0.459	−0.33	0.674
Pinto beans (n = 6)	0.73	0.100	0.87	0.024	0.13	0.811	−0.09	0.871	−0.32	0.530
Potato (n = 6)	0.49	0.324	0.55	0.257	0.38	0.452	0.16	0.756	0.17	0.742
Powdered milk (n = 4)	0.58	0.424	0.72	0.280	−0.23	0.772	−0.30	0.705	−0.46	0.536
Refined salt (n = 15)	0.20	0.483	0.12	0.670	0.55	0.034	0.39	0.150	0.33	0.231
Rolled oats (n = 9)	0.56	0.114	−0.30	0.439	−0.15	0.697	−0.03	0.944	−0.28	0.458
Sardine (n = 3)	−0.05	0.969	−0.95	0.201	0.27	0.827	0.59	0.597	0.43	0.719
Soy flour (n = 5)	0.59	0.290	0.71	0.176	0.89	0.045	0.94	0.016	0.84	0.074
Spinach (n = 4)	0.87	0.134	0.71	0.287	0.96	0.038	0.93	0.073	0.91	0.085
Supplement (n = 14)	0.65	0.012	0.06	0.842	0.17	0.564	−0.05	0.855	0.37	0.190
Wheat flour (n = 5)	0.03	0.966	−0.31	0.607	0.52	0.367	0.17	0.786	0.17	0.783
Whole wheat flour (n = 3)	0.00	0.999	0.66	0.545	0.49	0.673	0.95	0.210	0.84	0.368

n. the number of diets with this ingredient. Pearson correlation was used to determine coefficients.

**Table 7:** Correlation between ingredient inclusion in dry matter basis and heavy metal concentration in diets for cats, for ingredients with one or more moderately or highly positive correlation (r^2^ ≥ 0.5).

	Heavy metal									
	Cobalt (Co)		Mercury (Hg)		Lead (Pb)		Uranium (U)		Vanadium (V)	
	r^2^	p	r^2^	p	r^2^	p	r^2^	p	r^2^	p
Beef heart (n = 10)	0.06	0.870	0.56	0.093	−0.02	0.955	0.32	0.367	0.32	0.371
Beef liver (n = 7)	0.80	0.030	0.42	0.352	0.79	0.035	0.71	0.071	0.74	0.060
Beetroot (n = 3)	1.00	0.040	0.61	0.586	0.94	0.215	1.00	0.059	1.00	0.007
Bell pepper (n = 3)	1.00	0.057	0.89	0.302	1.00	0.043	1.00	0.005	1.00	0.020
Carrot (n = 8)	0.44	0.272	0.17	0.693	0.71	0.048	0.72	0.045	0.71	0.048
Celery (n = 3)	0.98	0.130	0.25	0.841	0.99	0.087	1.00	0.027	1.00	0.019
Chicken breast meat (n = 6)	0.81	0.050	0.41	0.417	0.86	0.030	0.84	0.037	0.76	0.080
Chicken gizzard (n = 3)	0.61	0.579	0.96	0.183	0.47	0.685	0.79	0.424	0.92	0.254
Collards (n = 4)	0.85	0.153	0.89	0.109	0.84	0.155	0.87	0.128	0.83	0.170
Eggshell meal (n = 5)	0.30	0.618	0.78	0.121	0.23	0.708	−0.23	0.708	−0.35	0.562
Squash (n = 7)	0.55	0.199	−0.24	0.605	0.48	0.276	0.61	0.143	0.55	0.204
Yam (n = 3)	0.95	0.204	0.86	0.344	0.99	0.103	0.92	0.260	0.96	0.185
Yogurt (n = 4)	0.44	0.558	−0.07	0.926	0.53	0.469	0.52	0.476	0.42	0.581

n. the number of diets with this ingredient. Pearson correlation was used to determine coefficients.

## Discussion

None of the diets supplied recommended levels of all nutrients evaluated, and more than 84.0% of diets presented three or more nutrients below recommendations for both dogs and cats. Nutrients with most levels below recommendations were calcium and potassium in recipes for dogs and iron and zinc in recipes for cats. As for heavy metals, levels of lead, cobalt, mercury, uranium, and vanadium were above MTLs.

There were differences in nutrient levels for recipes for dogs when considered comparisons with NRC^1^ and FEDIAF^2^ recommendations. This might be because these guidelines consider different diet characteristics for nutrient requirement tables, as the NRC^1^ considers diets with highly digestible and purified proteins, and energy requirement (ER) of 130 kcal/kg BW^0.75^, for animals with wide exercise opportunity or dogs in rural areas or housings with more than one animal.

However, recent studies point out that the energy expenditure of domiciled dogs may be lower than that of kennel dogs or dogs with an intense exercise routine. Bermingham *et al*.^44^ did a systematic review and meta-analysis on ER for domiciled adult dogs and observed mean maintenance energy requirement (MER) of 141 kcal/kg BW^0.75^ for active dogs and 95 kcal/kg BW^0.75^ for inactive dogs. Another study^45^ evaluated MER of client-owned dogs that underwent nutritional consultation in Munich, Germany, and the authors observed mean MER for healthy adult dogs of 98 kcal/kg BW^0.75^. A retrospective study^46^ conducted in Sao Paulo, Brazil, observed even lower MER of healthy adult dogs, with a mean value of 86.1 kcal/kg BW^0.75^. FEDIAF^2^ recommendations take into consideration this lower ER, and this may be one of the main reasons for the differences between results compared to NRC^1^ and FEDIAF^2^ recommendations in this study. As for cats, a study^47^ conducted in Munich, Germany, observed mean MER of 95 kcal/kg BW^0.67^, similar to recommendations of both NRC^1^ and FEDIAF^2^ of 100 kcal/kg BW^0.67^. However, many cats may need fewer energy^48,49^. Studies regarding indoor or neutered cats, which are an increasing population and may represent the majority of domesticated cats, suggest that the estimated average MER is 75 kcal/kg BW^0.67^ ^50,51^. Therefore, for the present study, lower MER was chosen to compare nutritional requirements.

Despite differences between nutritional requirement guidelines, the frequency of recipes with nutrient levels below recommendations was high, with more than 84.0% of diets presenting three or more nutrients below recommended levels. Clinical signs of nutritional deficiency are more promptly observed in the growth period due to higher nutritional demand. Adult animals, however, take longer to manifest nutrition-related diseases. One of the most commonly reported nutritional disease is nutritional secondary hyperparathyroidism (NSHP), caused by a calcium deficiency or low calcium:phosphorus ratio^22,23,52–54^. Results observed in this study, with a high frequency of recipes with calcium levels and calcium phosphorus ratio below recommendations, suggest the risk of developing NSHP if these recipes are consumed as a main diet. Furthermore, diets that had calcium levels below recommendations provided a median of only 20% of the nutrient requirement for dogs and up to 40% of the nutrient requirement for cats, increasing the risk of developing NSHP. Studies that evaluated mineral deficiencies in cats are scarce. Kienzle *et al*.^55^ assessed clinical signs of phosphorus deficiency in adult cats fed diets with calcium:phosphorus ratio of 4:1. Clinical signs observed were hemolytic anemia, difficulty to walk and metabolic acidosis. In the present study, 5 of the 25 diets for cats presented calcium:phosphorus ratio higher than 2:1, which could predispose animals to present clinical signs of phosphorus deficiency.

Protein and fat levels were below recommendations in some of the diets for both dogs and cats, especially when protein concentrations were compared to FEDIAF^2^. Most of the vegetarian and vegan diets for dogs and all vegetarian and vegan diets for cats were below FEDIAF^2^ recommended levels of protein. Protein deficiency should not be overlooked, as protein recommendations are made based on amino acid requirements^1^. Therefore, if protein concentration is below recommendation, it can be assumed that there may be one or more amino acids that do not supply daily recommendations. As for fat, several diets contained lean meat and no added fat source such as oils. This may be because of the humanization of pets, as fats are perceived as unhealthy ingredients for human health and therefore fat sources might be reduced in pet foods that are not properly balanced. Fat sources are an important energy source, as well as a source for essential fatty acids, and should be included to supply recommendations^1,2^.

Another nutrient with a high percentage of levels below recommendation was potassium. Deficiency of potassium in dogs may not cause clinical signs but may alter blood pressure and renal perfusion in the short term^56^. In cats, however, clinical signs of deficiency were observed in the long term. A study^27^ observed increased serum creatinine and potassium excretion in cats fed 3.4 g K/kg of diet in DM basis. When the same cats were fed a diet with 6.5 g K/kg of diet on a DM basis, clinical and laboratory signs were no longer present. Furthermore, Buffington *et al*.^26^ suggested that the intake of a diet containing 5 g K/kg of diet and more than 40% of protein in a DM basis for more than one year can lead to hypokalemia and renal dysfunction in cats. In the present study, the mean protein content of recipes for cats was 54% on a DM basis and mean potassium levels were 0.44%, which suggests that these diets could result in the previously mentioned clinical signs.

Zinc was also one of the minerals with a higher frequency of levels in the diets below recommended intake for dogs and cats. Zinc deficiency is caused by two main reasons: a genetic factor of some dog breeds, like Siberian Huskies; and low intake^1,21^. Clinical signs include alopecia and lesions on mucocutaneous junctions, and in histology, parakeratosis^21^. A study conducted in cats^25^ did not observe clinical signs in cats fed 15ppm of zinc, but animals fed this amount of zinc presented abnormal spermatogenesis that was not reversed after eight weeks of intake of 67ppm of this nutrient. There have been case reports of zinc deficiency in dogs that consumed diets with high amounts of phytate, present in cereals, and foods with high calcium content^24,57^. In the present study, the cereals that were used more often were white rice and brown rice, both of which contain low concentrations of phytate and therefore no significant interference in zinc absorption is expected^58^.

In recipes evaluated, especially in those for cats, iron was below recommendations for more than a third of the diets. Besides absolute low iron intake, it must be taken into consideration mineral interaction on absorption, such as calcium, phosphorus, zinc, and copper. Several recipes for both dogs and cats presented high levels of copper. There is no nutritional safe upper limit for copper, but high levels of this mineral may interfere in iron absorption and consequently reduce it, increasing the risk of signs of iron deficiency. The recommendations of adequate iron intake for cats are based on a study with kittens^59^, therefore more information on iron intake for adults is required to discuss with more precision the severity of consequences for cats that consume low amounts of iron.

It is important to state that, when recipes did not specify manufacturer or product, a commercial vitamin-mineral supplement designed for homemade diets was used, which only occurred in recipes for dogs. The product chosen is the only supplement specific for canine homemade diets available in major pet stores in Brazil. This arbitrary selection may have influenced the results of diets that contained unspecified supplements, but as the number of these diets is low (18.67%; n = 14/75), it may not have had an impact on results as a whole.

The risk of nutritional deficiencies can increase even more when the owner does not follow recipe instructions, already observed by previous studies. Johnson *et al*.^10^ observed that of owners that fed exclusively home-prepared diets for their dogs, 13.0% followed instructions as given. Another study^60^ observed that 50.0% of owners that fed homemade diets to dogs or cats did not follow instructions precisely, only 15.2% of interviewed owners had a scale to weigh ingredients, and 28.3% of owners admitted to not using supplementation prescribed. A more recent study^9^ observed that of the 110 owners that took part in the research, 60.0% made some kind of change in the prescribed diet, either changing type of meat or adding a different ingredient. Furthermore, 35.1% admitted not to follow properly the amounts of ingredients. These alterations to the diet can lead to decreased intake of some nutrients, and also change energy intake, which can cause nutritional diseases in the long term o even aggravate pre-existing clinical diseases.

A possible limiting factor of this study was that water used for cooking was not analyzed for heavy metal contamination. However, all recipes for cats presented vanadium above MTL, including raw diets, and raw recipes for dogs and cats presented uranium levels above MTL. Therefore, the water used for cooking does not seem to be an influencing factor. Some ingredients, however, were positively correlated with heavy metals that were above MTLs. Despite *p* values ≤ 0.05 in some of the correlations, moderate and high coefficients may still be considered, as the number of diets that contained each ingredient was low, and this may have influenced the *p* values.

Lead concentrations were above MTL in more than a quarter of the recipes analyzed. This metal may be present in the environment by fossil fuel burning, like coal and petroleum derivatives, and by pesticide use. In the past, the addition of tetraethyl lead (TEL) was permitted to boost the octane rating of fuels. Despite being prohibited in Brazil in 1989, soil contamination can be attributed to its past use^61^. Signs of lead poisoning vary and may include signs of neurotoxicity, hematologic dysfunction, renal dysfunction, arterial hypertension, and carcinogenesis^62^. As for uranium, more than 90.0% of all recipes presented concentrations above MTLs, and main sources of contamination are soil and water^63^. Brazil has the 5^th^ greatest uranium reserve, located mainly in states of Bahia, Minas Gerais and Sao Paulo^64^, which could justify its higher amounts in foods acquired from these regions, as is the case of this study. Uranium toxicity can lead to signs of renal dysfunction because it accumulates in the tubular epithelial cells and causes necrosis^63^.

Mercury was present in high levels in more than 70.0% of recipes. Intoxication signs are mainly neurological, and histological evaluation may point to neuronal degeneration, loss of astrocytes and glial proliferation on cortical cerebral portion^37^. One study^65^ evaluated mercury concentrations in dry and wet pet food in the United States and observed that foods with the highest amounts of this metal contained tuna, shrimp and salmon. Charbonneau *et al*.^66^ evaluated chronic mercury consumption by cats and observed neurobehavioral changes after intake of 0.046 mg of mercury/kg BW/day in contaminated fish. When intake was 0.074 mg of mercury/kg BW/day, cats presented neurological signs including seizures. This study was the base for establishing mercury MTL for cats and was extrapolated for dogs.

Cobalt levels were increased in only four of the recipes and did not exceed 20.0% of MTLs. Vanadium concentrations, on the other hand, were above MTLs in more than half of the recipes, exceeding by four times recommended values in some cases. There are few studies about safe upper limits for these two elements, and their MTLs are based on research in other species^37^. FDA^36^ considers values that are ten times lower than the most sensitive mammal according to NRC^37^ for cobalt, uranium, and vanadium, to extrapolate between species. Therefore, if dogs and cats are less sensitive than current MTLs proposed, the safe upper limit may be higher and this would reduce the number of recipes that exceed the limit.

A possible limiting factor of this study was that water used for cooking was not analyzed for heavy metal contamination. However, all recipes for cats presented vanadium above MTL, including raw diets, and raw recipes for dogs and cats presented uranium levels above MTL. Therefore, the water used for cooking does not seem to be an influencing factor.

There is a limited number of studies that evaluated heavy metal concentration in pet foods and its potential risks to dogs and cats. Davies *et al*.^67^ evaluated dry and wet foods for dogs and cats for their mineral and heavy metal composition. Unlike the present study, lead and uranium levels were below detection limits established by the European Union (EU). However, EU recommendations^68^ are for foods with moisture around 12.0%, so in the present study, results were compared to FDA^36^ and NRC^37^ recommendations because moisture for most recipes evaluated was higher than 65.0%. Another study^35^ conducted in Brazil evaluated 95 samples of dry food for dogs and puppies. Concentrations of arsenic, cadmium, and mercury were not considered a risk when compared to maximum safe levels for humans according to *Codex Alimentatius*^69^, and concentrations of aluminum, antimony, and uranium were considered high according to the same standards.

## Conclusion

Home-prepared diets, when not formulated properly, offer risk to the health status of dogs and cats. In the present study, none of the recipes evaluated met all recommendations of protein, fat, and minerals. It can be concluded that the formulation of home-prepared diets needs to be provided by a trained professional to minimize the risk of nutritional imbalances, to ensure better health, quality of life and increase the lifespan of dogs and cats. Regarding heavy metals, fourteen of the fifteen elements were detectable, and concentrations of cobalt, lead, mercury, uranium, and vanadium were the only ones above MTLs. The real implications of these results have not yet been totally elucidated, and studies that considered chronic heavy metal intake are necessary to better understand safe limits for dogs and cats.

## Supplementary information

Supplementary Info

## Data Availability

The datasets generated during and/or analyzed in the current study are available from the corresponding author on reasonable request.
