# Supplementary material for: Concentrations of macronutrients, minerals and heavy metals in home-prepared diets for adult dogs and cats
Source: Sci Rep. 2019 Sep 10;9:13058. doi: 10.1038/s41598-019-49087-z (PMC6736975; doi:10.1038/s41598-019-49087-z)
Supplement: Supplementary file 1 — Supplementary Info [file 41598_2019_49087_MOESM1_ESM.docx]

**Concentrations of macronutrients, minerals and heavy metals in home-prepared diets for adult dogs and cats**

**Vivian Pedrinelli^1^, Rafael Vessecchi Amorim Zafalon^2^, Roberta Bueno Ayres Rodrigues^2^, Mariana Pamplona Perini^2^, Renata Maria Consentino Conti^2^, Thiago Henrique Annibale Vendramini^2^, Júlio César de Carvalho Balieiro^2^ & Márcio Antonio Brunetto^2^***

^1^School of Veterinary Medicine and Animal Science, University of Sao Paulo (USP) – Department of Medical Clinic, São Paulo, postcode 13690-970, Brazil.

^2^School of Veterinary Medicine and Animal Science, University of Sao Paulo (USP) – Department of Animal Nutrition and Production, Pirassununga, postcode 13635-900, Brazil.

Correspondence and requests for materials should be addressed to M.A.B. (email: [mabrunetto@usp.br](mailto:mabrunetto@usp.br))

Supplementary Table S1 – Results of the analyses of 75 homemade diets for healthy adult dogs per 1000 kcal.

|  | **Dry matter (%)** | **Metabolizable energy**  **(kcal/g)** | **Crude protein**  **(g)** | **Fat**  **(g)** | **Crude fiber**  **(g)** | **Ash (g)** | **Calcium**  **(g)** | **Phosphorus (g)** | **Ca:P ratio** | **Potassium (g)** | **Magnesium (g)** | **Sodium**  **(g)** | **Copper (mg)** | **Iron (mg)** | **Manganese (mg)** | **Zinc (mg)** |
| --- | --- | --- | --- | --- | --- | --- | --- | --- | --- | --- | --- | --- | --- | --- | --- | --- |
| NRC | - | - | 25.00 | 13.80 | - | - | 1.00 | 0.75 | - | 1.00 | 0.15 | 0.20 | 1.50 | 7.50 | 1.20 | 15.00 |
| FEDIAF | - | - | 52.10 | 13.75 | - | - | 1.45 | 1.16 | 1(min)/2(max) | 1.45 | 0.20 | 0.29 | 2.08 | 10.40 | 1.67 | 20.80 |
| Diet 1 | 36.87 | 4.03 | 65.35 | 16.47 | 3.63 | 15.35 | 2.93 | 0.66 | 4.47 | 0.55 | 0.12 | 1.49 | 3.15 | 11.10 | 1.49 | 15.04 |
| Diet 2 | 29.37 | 4.02 | 71.29 | 9.18 | 3.56 | 6.67 | 1.37 | 1.14 | 1.20 | 0.25 | 0.13 | 0.08 | 18.33 | 3.93 | 2.84 | 26.35 |
| Diet 3 | 26.71 | 4.18 | 82.27 | 21.28 | 4.93 | 10.76 | 1.04 | 1.42 | 0.73 | 0.86 | 0.34 | 0.79 | 26.01 | 32.40 | 7.85 | 32.92 |
| Diet 4 | 27.54 | 4.89 | 123.24 | 49.43 | 3.87 | 12.61 | 1.50 | 1.43 | 1.05 | 0.76 | 0.20 | 0.63 | 16.03 | 13.10 | 0.52 | 8.75 |
| Diet 5 | 55.55 | 4.11 | 82.56 | 38.94 | 24.60 | 17.63 | 4.53 | 0.77 | 5.85 | 0.73 | 0.16 | 1.07 | 23.06 | 9.57 | 0.10 | 21.07 |
| Diet 6 | 16.27 | 3.87 | 122.26 | 10.07 | 10.98 | 9.87 | 0.36 | 1.17 | 0.31 | 1.81 | 0.36 | 0.22 | 5.89 | 12.24 | 4.50 | 8.28 |
| Diet 7 | 28.43 | 5.08 | 113.49 | 56.76 | 4.84 | 12.80 | 1.49 | 1.56 | 0.96 | 1.08 | 0.24 | 1.10 | 5.92 | 17.62 | 0.59 | 10.29 |
| Diet 8 | 25.84 | 4.24 | 100.87 | 33.23 | 12.12 | 15.26 | 2.12 | 0.77 | 2.74 | 1.56 | 0.16 | 1.18 | 9.50 | 10.35 | 0.87 | 19.58 |
| Diet 9 | 21.75 | 4.14 | 122.82 | 21.12 | 6.64 | 11.15 | 1.16 | 1.03 | 1.12 | 1.26 | 0.26 | 1.09 | 3.81 | 6.52 | 2.27 | 4.91 |
| Diet 10 | 28.15 | 4.53 | 77.08 | 40.98 | 7.70 | 14.19 | 2.36 | 1.16 | 2.03 | 1.19 | 0.19 | 1.05 | 5.27 | 9.22 | 0.70 | 5.26 |
| Diet 11 | 43.5 | 4.04 | 52.18 | 39.61 | 20.20 | 26.86 | 6.86 | 2.22 | 3.08 | 0.77 | 0.12 | 1.81 | 2.35 | 4.16 | 0.96 | 1.80 |
| Diet 12 | 35.25 | 5.00 | 60.67 | 52.42 | 12.21 | 3.16 | 0.10 | 0.53 | 0.19 | 0.82 | 0.07 | 0.26 | 2.38 | 4.48 | 1.04 | 6.78 |
| Diet 13 | 34.85 | 3.97 | 100.19 | 16.34 | 8.60 | 14.00 | 1.26 | 2.19 | 0.57 | 1.56 | 0.32 | 0.78 | 4.64 | 36.94 | 15.66 | 20.79 |
| Diet 14 | 51.47 | 4.05 | 85.86 | 16.26 | 5.41 | 11.98 | 1.06 | 2.07 | 0.51 | 1.31 | 0.36 | 0.12 | 22.80 | 18.53 | 17.10 | 18.92 |
| Diet 15 | 24.85 | 4.31 | 102.87 | 26.38 | 5.55 | 9.64 | 0.26 | 0.89 | 0.29 | 1.51 | 0.12 | 0.27 | 4.32 | 10.77 | 0.39 | 10.90 |
| Diet 16 | 33.69 | 3.80 | 46.66 | 8.39 | 8.20 | 15.14 | 3.34 | 1.17 | 2.85 | 1.39 | 0.32 | 0.10 | 8.70 | 20.74 | 8.27 | 13.70 |
| Diet 17 | 31.86 | 4.36 | 93.51 | 38.81 | 6.40 | 21.47 | 0.85 | 0.64 | 1.33 | 1.17 | 0.07 | 1.44 | 10.71 | 17.43 | 2.17 | 7.01 |
| Diet 18 | 39.87 | 4.54 | 96.57 | 34.18 | 2.01 | 11.11 | 1.01 | 2.02 | 0.50 | 1.16 | 0.19 | 0.44 | 3.88 | 25.48 | 3.89 | 13.26 |
| Diet 19 | 36.84 | 4.22 | 51.89 | 17.57 | 2.54 | 6.57 | 0.92 | 0.69 | 1.34 | 0.58 | 0.14 | 0.10 | 11.81 | 12.81 | 3.89 | 10.40 |
| Diet 20 | 53.62 | 4.58 | 75.61 | 40.31 | 8.48 | 10.38 | 0.98 | 1.07 | 0.92 | 0.95 | 0.16 | 0.78 | 5.20 | 10.09 | 4.49 | 10.04 |
| Diet 21 | 21.65 | 4.39 | 120.76 | 33.26 | 4.33 | 15.23 | 1.07 | 2.39 | 0.45 | 1.43 | 0.22 | 0.34 | 8.16 | 26.26 | 5.11 | 27.57 |
| Diet 22 | 25.88 | 4.93 | 67.20 | 57.25 | 7.50 | 16.72 | 3.59 | 1.05 | 3.42 | 1.41 | 0.13 | 0.19 | 6.30 | 48.23 | 4.29 | 26.37 |
| Diet 23 | 37.32 | 4.57 | 73.42 | 33.01 | 3.17 | 6.98 | 0.97 | 1.17 | 0.83 | 0.31 | 0.28 | 0.10 | 17.51 | 24.56 | 6.49 | 21.03 |

(Continues)

|  | **Dry matter (%)** | **Metabolizable energy**  **(kcal/g)** | **Crude protein**  **(g)** | **Fat**  **(g)** | **Crude fiber**  **(g)** | **Ash (g)** | **Calcium**  **(g)** | **Phosphorus (g)** | **Ca:P ratio** | **Potassium (g)** | **Magnesium (g)** | **Sodium**  **(g)** | **Copper (mg)** | **Iron (mg)** | **Manganese (mg)** | **Zinc (mg)** |
| --- | --- | --- | --- | --- | --- | --- | --- | --- | --- | --- | --- | --- | --- | --- | --- | --- |
| NRC | - | - | 25.00 | 13.80 | - | - | 1.00 | 0.75 | - | 1.00 | 0.15 | 0.20 | 1.50 | 7.50 | 1.20 | 15.00 |
| FEDIAF | - | - | 52.10 | 13.75 | - | - | 1.45 | 1.16 | 1(min)/2(max) | 1.45 | 0.20 | 0.29 | 2.08 | 10.40 | 1.67 | 20.80 |
| Diet 24 | 39.32 | 4.60 | 70.51 | 47.45 | 19.36 | 7.24 | 0.05 | 0.07 | 0.78 | 0.06 | 0.01 | 0.03 | 0.73 | 0.92 | 0.23 | 1.20 |
| Diet 25 | 30.89 | 3.02 | 134.19 | 15.72 | 20.96 | 80.28 | 0.07 | 0.10 | 0.76 | 0.09 | 0.01 | 0.05 | 1.02 | 1.21 | 0.29 | 1.65 |
| Diet 26 | 24.22 | 3.93 | 77.41 | 7.19 | 1.73 | 11.41 | 0.04 | 0.05 | 0.94 | 0.07 | 0.01 | 0.04 | 0.81 | 0.49 | 0.12 | 0.88 |
| Diet 27 | 33.39 | 4.20 | 47.29 | 16.23 | 1.14 | 7.16 | 1.24 | 1.51 | 0.82 | 0.23 | 0.28 | 0.27 | 11.31 | 34.10 | 7.46 | 23.29 |
| Diet 28 | 28.35 | 4.19 | 128.69 | 17.03 | 3.34 | 6.78 | 0.04 | 0.68 | 0.06 | 0.30 | 0.13 | 0.07 | 1.83 | 1.95 | 1.30 | 2.63 |
| Diet 29 | 20.79 | 4.24 | 123.72 | 23.01 | 5.62 | 9.16 | 0.13 | 1.22 | 0.11 | 0.63 | 0.14 | 0.21 | 8.44 | 21.10 | 1.05 | 10.60 |
| Diet 30 | 21.45 | 4.30 | 102.02 | 25.42 | 6.54 | 8.06 | 0.19 | 1.30 | 0.15 | 1.07 | 0.20 | 0.21 | 13.23 | 13.35 | 0.61 | 17.97 |
| Diet 31 | 27.69 | 4.01 | 124.14 | 16.76 | 4.83 | 15.19 | 3.68 | 0.81 | 4.52 | 1.02 | 0.19 | 0.30 | 8.31 | 8.56 | 0.79 | 31.40 |
| Diet 32 | 25.77 | 4.25 | 99.21 | 24.92 | 3.69 | 12.60 | 2.87 | 0.74 | 3.89 | 0.81 | 0.16 | 0.22 | 14.71 | 10.24 | 1.02 | 19.94 |
| Diet 33 | 29.17 | 4.34 | 126.08 | 25.29 | 2.86 | 9.24 | 0.09 | 1.21 | 0.07 | 1.07 | 0.17 | 0.80 | 22.48 | 21.88 | 0.91 | 23.82 |
| Diet 34 | 22.55 | 4.28 | 73.60 | 22.62 | 5.28 | 6.44 | 0.17 | 1.04 | 0.16 | 0.84 | 0.13 | 1.04 | 10.72 | 9.36 | 1.58 | 14.91 |
| Diet 35 | 29.22 | 4.11 | 62.56 | 8.88 | 0.68 | 3.75 | 0.17 | 0.74 | 0.23 | 0.39 | 0.12 | 0.11 | 10.18 | 4.02 | 1.73 | 4.60 |
| Diet 36 | 25.14 | 4.23 | 94.33 | 19.18 | 5.89 | 4.64 | 0.10 | 0.95 | 0.10 | 0.47 | 0.16 | 0.26 | 3.80 | 4.01 | 1.57 | 5.47 |
| Diet 37 | 25.87 | 3.81 | 79.74 | 5.06 | 9.75 | 8.70 | 0.34 | 1.37 | 0.25 | 0.70 | 0.42 | 0.38 | 6.68 | 15.72 | 5.38 | 10.23 |
| Diet 38 | 15.65 | 4.06 | 110.13 | 14.92 | 7.81 | 7.07 | 0.14 | 1.02 | 0.13 | 0.58 | 0.44 | 0.10 | 7.76 | 6.11 | 5.97 | 7.54 |
| Diet 39 | 22.91 | 3.86 | 17.36 | 0.39 | 3.57 | 5.67 | 0.14 | 0.36 | 0.40 | 0.72 | 0.08 | 0.06 | 19.47 | 3.60 | 1.57 | 2.83 |
| Diet 40 | 28.30 | 4.06 | 95.29 | 10.62 | 4.95 | 4.81 | 0.06 | 1.36 | 0.04 | 0.43 | 0.34 | 0.09 | 13.59 | 6.06 | 4.14 | 5.65 |
| Diet 41 | 26.05 | 4.38 | 109.69 | 26.66 | 4.91 | 6.83 | 0.09 | 1.32 | 0.07 | 0.62 | 0.14 | 0.19 | 15.53 | 13.04 | 1.12 | 22.88 |
| Diet 42 | 31.22 | 5.25 | 37.09 | 53.63 | 2.55 | 4.87 | 0.87 | 0.55 | 1.57 | 0.22 | 0.09 | 0.16 | 3.96 | 5.59 | 2.32 | 6.56 |
| Diet 43 | 27.80 | 4.00 | 52.39 | 22.26 | 19.73 | 8.15 | 0.40 | 1.45 | 0.27 | 0.88 | 0.42 | 0.12 | 15.62 | 15.62 | 4.53 | 7.61 |
| Diet 44 | 34.12 | 3.85 | 64.48 | 25.99 | 31.13 | 11.23 | 1.06 | 1.03 | 1.02 | 1.27 | 0.36 | 0.31 | 6.75 | 13.74 | 2.20 | 8.76 |
| Diet 45 | 22.65 | 5.46 | 51.26 | 79.32 | 21.43 | 10.72 | 2.74 | 0.79 | 3.47 | 0.63 | 0.19 | 0.33 | 4.58 | 9.80 | 3.60 | 7.93 |
| Diet 46 | 25.35 | 4.14 | 64.16 | 30.64 | 18.19 | 11.95 | 1.19 | 1.32 | 0.90 | 1.01 | 0.30 | 0.70 | 23.12 | 11.15 | 1.77 | 6.18 |
| Diet 47 | 37.09 | 4.20 | 44.95 | 26.77 | 15.47 | 5.92 | 0.20 | 1.06 | 0.19 | 0.63 | 0.30 | 0.05 | 26.44 | 7.14 | 6.64 | 6.52 |

(Continues)

|  | **Dry matter (%)** | **Metabolizable energy**  **(kcal/g)** | **Crude protein**  **(g)** | **Fat**  **(g)** | **Crude fiber**  **(g)** | **Ash (g)** | **Calcium**  **(g)** | **Phosphorus (g)** | **Ca:P ratio** | **Potassium (g)** | **Magnesium (g)** | **Sodium**  **(g)** | **Copper (mg)** | **Iron (mg)** | **Manganese (mg)** | **Zinc (mg)** |
| --- | --- | --- | --- | --- | --- | --- | --- | --- | --- | --- | --- | --- | --- | --- | --- | --- |
| NRC | - | - | 25.00 | 13.80 | - | - | 1.00 | 0.75 | - | 1.00 | 0.15 | 0.20 | 1.50 | 7.50 | 1.20 | 15.00 |
| FEDIAF | - | - | 52.10 | 13.75 | - | - | 1.45 | 1.16 | 1(min)/2(max) | 1.45 | 0.20 | 0.29 | 2.08 | 10.40 | 1.67 | 20.80 |
| Diet 48 | 26.14 | 4.47 | 110.41 | 50.05 | 27.81 | 8.48 | 0.14 | 0.95 | 0.15 | 0.60 | 0.17 | 0.89 | 4.77 | 9.60 | 1.10 | 24.66 |
| Diet 49 | 31.78 | 4.65 | 130.65 | 48.88 | 16.44 | 9.90 | 0.58 | 1.11 | 0.52 | 0.70 | 0.15 | 0.46 | 10.04 | 11.66 | 0.57 | 23.99 |
| Diet 50 | 17.19 | 4.13 | 92.03 | 24.44 | 13.59 | 9.13 | 0.31 | 1.16 | 0.26 | 1.05 | 0.16 | 0.65 | 9.18 | 4.43 | 1.16 | 5.43 |
| Diet 51 | 24.04 | 3.97 | 119.98 | 8.74 | 6.42 | 6.35 | 0.18 | 1.24 | 0.14 | 0.74 | 0.18 | 0.13 | 2.93 | 2.44 | 1.18 | 5.70 |
| Diet 52 | 41.08 | 4.22 | 65.36 | 24.58 | 12.39 | 5.16 | 0.10 | 0.93 | 0.11 | 0.51 | 0.19 | 0.38 | 22.72 | 8.34 | 2.33 | 18.42 |
| Diet 53 | 29.32 | 4.16 | 125.11 | 31.00 | 14.09 | 15.15 | 3.40 | 1.65 | 2.06 | 1.07 | 0.37 | 0.27 | 64.51 | 39.76 | 7.01 | 47.13 |
| Diet 54 | 36.99 | 4.42 | 123.65 | 30.69 | 6.18 | 8.49 | 0.25 | 1.38 | 0.18 | 0.74 | 0.29 | 0.22 | 18.44 | 18.80 | 6.25 | 25.34 |
| Diet 55 | 17.07 | 3.78 | 37.98 | 6.88 | 14.37 | 8.89 | 0.38 | 1.14 | 0.33 | 0.92 | 0.42 | 0.07 | 4.61 | 11.44 | 8.63 | 8.57 |
| Diet 56 | 65.66 | 4.37 | 67.78 | 28.67 | 6.75 | 8.06 | 0.89 | 1.64 | 0.54 | 0.69 | 0.25 | 0.43 | 4.07 | 12.45 | 8.66 | 15.71 |
| Diet 57 | 24.86 | 4.06 | 157.01 | 13.94 | 4.85 | 8.79 | 0.09 | 1.35 | 0.07 | 0.83 | 0.30 | 0.14 | 9.84 | 6.14 | 2.33 | 6.65 |
| Diet 58 | 60.32 | 3.98 | 46.70 | 10.74 | 9.93 | 4.95 | 0.18 | 1.29 | 0.14 | 0.44 | 0.37 | 0.17 | 2.47 | 4.70 | 5.18 | 10.05 |
| Diet 59 | 64.62 | 4.13 | 40.13 | 15.16 | 9.23 | 1.91 | 0.09 | 0.50 | 0.19 | 0.23 | 0.08 | 0.06 | 15.76 | 1.17 | 1.67 | 3.76 |
| Diet 60 | 91.05 | 4.36 | 43.95 | 27.45 | 5.94 | 7.85 | 0.45 | 0.92 | 0.49 | 0.60 | 0.26 | 0.66 | 2.63 | 9.67 | 8.16 | 7.99 |
| Diet 61 | 23.16 | 4.74 | 94.55 | 47.63 | 12.67 | 7.63 | 0.18 | 1.31 | 0.14 | 0.91 | 0.19 | 0.26 | 5.91 | 4.24 | 0.27 | 3.73 |
| Diet 62 | 69.11 | 4.25 | 71.17 | 27.55 | 11.42 | 8.04 | 0.65 | 1.43 | 0.45 | 0.83 | 0.31 | 0.31 | 4.60 | 10.86 | 11.04 | 16.74 |
| Diet 63 | 82.76 | 4.51 | 40.64 | 55.33 | 35.85 | 4.87 | 0.34 | 0.94 | 0.36 | 0.38 | 0.18 | 0.33 | 1.19 | 10.22 | 6.99 | 8.31 |
| Diet 64 | 47.47 | 4.71 | 79.26 | 75.65 | 52.06 | 4.65 | 0.31 | 0.89 | 0.34 | 0.48 | 0.11 | 0.28 | 0.88 | 8.31 | 0.61 | 14.14 |
| Diet 65 | 32.56 | 4.14 | 112.24 | 25.18 | 13.81 | 9.10 | 0.12 | 1.20 | 0.10 | 0.86 | 0.31 | 0.93 | 1.41 | 8.50 | 2.32 | 8.18 |
| Diet 66 | 22.05 | 4.65 | 114.71 | 46.27 | 12.83 | 9.91 | 0.24 | 1.40 | 0.17 | 1.28 | 0.22 | 0.41 | 1.30 | 11.83 | 1.73 | 25.91 |
| Diet 67 | 63.85 | 3.98 | 27.24 | 7.84 | 6.63 | 4.42 | 0.06 | 1.13 | 0.05 | 0.34 | 0.38 | 0.10 | 1.79 | 5.92 | 6.04 | 6.11 |
| Diet 68 | 31.40 | 4.18 | 135.39 | 20.45 | 7.63 | 7.05 | 0.21 | 1.39 | 0.15 | 0.55 | 0.27 | 0.42 | 1.47 | 5.31 | 1.76 | 7.34 |
| Diet 69 | 25.61 | 4.00 | 117.99 | 17.42 | 14.35 | 7.35 | 0.09 | 1.12 | 0.08 | 0.69 | 0.15 | 0.67 | 1.07 | 2.67 | 1.10 | 7.21 |
| Diet 70 | 28.94 | 4.12 | 154.42 | 15.15 | 5.90 | 5.75 | 0.14 | 1.15 | 0.12 | 0.66 | 0.25 | 0.12 | 1.11 | 6.83 | 2.74 | 7.01 |
| Diet 71 | 18.35 | 4.40 | 112.62 | 33.83 | 8.09 | 11.38 | 0.36 | 1.37 | 0.26 | 1.19 | 0.42 | 0.23 | 2.47 | 15.39 | 4.88 | 25.65 |

(Continues)

(Conclusion)

|  | **Dry matter (%)** | **Metabolizable energy**  **(kcal/g)** | **Crude protein**  **(g)** | **Fat**  **(g)** | **Crude fiber**  **(g)** | **Ash (g)** | **Calcium**  **(g)** | **Phosphorus (g)** | **Ca:P ratio** | **Potassium (g)** | **Magnesium (g)** | **Sodium**  **(g)** | **Copper (mg)** | **Iron (mg)** | **Manganese (mg)** | **Zinc (mg)** |
| --- | --- | --- | --- | --- | --- | --- | --- | --- | --- | --- | --- | --- | --- | --- | --- | --- |
| NRC | - | - | 25.00 | 13.80 | - | - | 1.00 | 0.75 | - | 1.00 | 0.15 | 0.20 | 1.50 | 7.50 | 1.20 | 15.00 |
| FEDIAF | - | - | 52.10 | 13.75 | - | - | 1.45 | 1.16 | 1(min)/2(max) | 1.45 | 0.20 | 0.29 | 2.08 | 10.40 | 1.67 | 20.80 |
| Diet 72 | 22.37 | 4.43 | 113.76 | 32.26 | 5.62 | 10.59 | 0.32 | 1.39 | 0.23 | 1.25 | 0.48 | 0.10 | 3.12 | 9.86 | 4.67 | 7.62 |
| Diet 73 | 27.46 | 4.45 | 120.70 | 31.64 | 4.18 | 10.25 | 0.31 | 1.40 | 0.22 | 1.34 | 0.46 | 0.10 | 2.67 | 9.65 | 5.02 | 8.15 |
| Diet 74 | 20.15 | 4.18 | 75.16 | 29.01 | 16.11 | 9.13 | 0.66 | 0.97 | 0.68 | 0.68 | 0.24 | 0.23 | 1.67 | 12.84 | 2.67 | 5.13 |
| Diet 75 | 46.37 | 4.84 | 51.12 | 49.57 | 10.98 | 7.78 | 0.19 | 1.08 | 0.18 | 0.57 | 0.26 | 0.94 | 1.74 | 9.04 | 5.71 | 7.25 |

Ca. calcium. P. phosphorus.

Supplementary Table S2 – Results of the analyses of 25 homemade diets for healthy adult cats per 1000 kcal.

|  | **Dry matter (%)** | **Metabolizable energy**  **(kcal/g)** | **Crude protein**  **(g)** | **Fat**  **(g)** | **Crude fiber**  **(g)** | **Ash (g)** | **Calcium**  **(g)** | **Phosphorus (g)** | **Ca:P ratio** | **Potassium (g)** | **Magnesium (g)** | **Sodium**  **(g)** | **Copper (mg)** | **Iron (mg)** | **Manganese (mg)** | **Zinc (mg)** |
| --- | --- | --- | --- | --- | --- | --- | --- | --- | --- | --- | --- | --- | --- | --- | --- | --- |
| NRC | - | - | 50.00 | 22.50 | - | - | 0.72 | 0.64 | - | 1.30 | 0.10 | 0.17 | 1.20 | 20.00 | 1.20 | 18.50 |
| FEDIAF | - | - | 83.30 |  | - | - | 1.97 | 1.67 | 1(min)/2(max) | 2.00 | 0.13 | 0.25 | 0.1.67 | 26.70 | 1.67 | 25.00 |
| Diet 1 | 36.87 | 3.99 | 65.89 | 16.61 | 3.66 | 15.48 | 2.95 | 0.66 | 4.47 | 0.55 | 0.12 | 1.50 | 3.18 | 11.20 | 1.50 | 15.16 |
| Diet 2 | 29.37 | 4.00 | 71.62 | 9.22 | 3.57 | 6.70 | 1.37 | 1.15 | 1.20 | 0.25 | 0.13 | 0.08 | 18.42 | 3.96 | 2.85 | 26.47 |
| Diet 3 | 21.68 | 4.19 | 179.00 | 28.21 | 4.25 | 16.34 | 2.41 | 1.67 | 1.44 | 1.57 | 0.25 | 1.11 | 13.33 | 23.65 | 0.84 | 15.85 |
| Diet 4 | 21.38 | 4.93 | 120.60 | 58.37 | 12.83 | 11.57 | 1.68 | 1.06 | 1.59 | 1.15 | 0.17 | 0.95 | 12.38 | 25.29 | 0.64 | 24.02 |
| Diet 5 | 26.48 | 4.13 | 192.21 | 23.69 | 2.98 | 15.63 | 1.70 | 0.18 | 9.51 | 1.58 | 0.26 | 1.14 | 30.78 | 18.31 | 0.56 | 17.93 |
| Diet 6 | 33.22 | 4.72 | 114.56 | 51.49 | 3.13 | 16.58 | 3.28 | 1.96 | 1.67 | 1.16 | 0.29 | 1.11 | 20.64 | 18.30 | 1.05 | 10.56 |
| Diet 7 | 28.09 | 4.41 | 175.76 | 34.44 | 3.04 | 12.40 | 0.30 | 1.52 | 0.20 | 1.22 | 0.21 | 0.53 | 16.37 | 16.61 | 0.64 | 14.46 |
| Diet 8 | 33.14 | 4.92 | 137.11 | 50.44 | 22.12 | 10.46 | 1.31 | 0.92 | 1.42 | 1.11 | 0.16 | 1.01 | 5.94 | 17.56 | 0.21 | 29.66 |
| Diet 9 | 27.82 | 4.00 | 207.17 | 18.66 | 5.97 | 14.84 | 1.90 | 1.71 | 1.11 | 1.56 | 0.23 | 1.00 | 20.77 | 14.67 | 0.33 | 14.28 |
| Diet 10 | 34.22 | 4.70 | 123.68 | 48.64 | 4.06 | 13.24 | 1.89 | 1.85 | 1.03 | 1.17 | 0.24 | 1.17 | 6.90 | 13.76 | 0.76 | 8.26 |
| Diet 11 | 35.57 | 4.50 | 105.03 | 37.55 | 3.09 | 11.49 | 1.74 | 1.64 | 1.06 | 0.72 | 0.24 | 0.71 | 5.04 | 10.16 | 0.89 | 12.67 |
| Diet 12 | 22.64 | 4.33 | 168.44 | 34.83 | 4.25 | 16.09 | 4.05 | 1.25 | 3.24 | 0.87 | 0.25 | 0.35 | 4.87 | 7.56 | 0.48 | 7.53 |
| Diet 13 | 39.13 | 4.30 | 133.07 | 48.63 | 23.69 | 13.46 | 2.09 | 0.97 | 2.15 | 0.74 | 0.19 | 0.40 | 2.96 | 12.24 | 0.29 | 35.29 |
| Diet 14 | 33.05 | 4.82 | 99.98 | 52.10 | 3.30 | 12.58 | 2.16 | 1.79 | 1.21 | 0.53 | 0.24 | 0.71 | 5.77 | 9.86 | 1.30 | 9.50 |
| Diet 15 | 28.40 | 3.97 | 186.66 | 21.99 | 4.49 | 22.42 | 5.36 | 2.10 | 2.55 | 0.99 | 0.28 | 0.31 | 52.26 | 20.38 | 1.10 | 18.79 |
| Diet 16 | 21.12 | 4.18 | 96.94 | 26.89 | 12.07 | 7.66 | 0.12 | 1.28 | 0.09 | 1.05 | 0.28 | 0.21 | 6.26 | 11.11 | 2.44 | 21.28 |
| Diet 17 | 18.70 | 4.03 | 110.99 | 23.45 | 15.72 | 8.58 | 0.24 | 0.75 | 0.32 | 1.66 | 0.26 | 1.34 | 1.30 | 5.57 | 1.43 | 4.69 |
| Diet 18 | 32.34 | 4.72 | 154.70 | 41.47 | 6.76 | 12.28 | 1.02 | 1.39 | 0.73 | 0.97 | 0.19 | 0.73 | 22.44 | 18.09 | 0.33 | 30.94 |
| Diet 19 | 23.15 | 6.33 | 60.67 | 87.88 | 22.05 | 5.45 | 0.40 | 0.78 | 0.51 | 0.49 | 0.08 | 0.57 | 28.99 | 7.33 | 0.35 | 6.33 |
| Diet 20 | 27.72 | 4.74 | 41.78 | 56.11 | 18.75 | 5.29 | 0.24 | 0.80 | 0.30 | 0.44 | 0.26 | 0.24 | 3.46 | 8.21 | 3.88 | 6.72 |
| Diet 21 | 18.63 | 3.88 | 107.39 | 16.72 | 8.04 | 18.37 | 2.61 | 1.61 | 1.62 | 1.15 | 0.41 | 0.25 | 1.68 | 11.11 | 13.14 | 23.39 |
| Diet 22 | 26.03 | 4.16 | 210.53 | 17.65 | 1.66 | 8.33 | 0.09 | 1.60 | 0.06 | 0.82 | 0.24 | 0.20 | 0.87 | 6.67 | 0.02 | 11.54 |
| Diet 23 | 6.20 | 3.28 | 42.45 | 11.43 | 37.88 | 29.75 | 1.14 | 1.61 | 0.71 | 2.13 | 0.52 | 0.16 | 3.34 | 18.19 | 15.88 | 18.36 |

(Continues)

(Conclusion)

|  | **Dry matter (%)** | **Metabolizable energy**  **(kcal/g)** | **Crude protein**  **(g)** | **Fat**  **(g)** | **Crude fiber**  **(g)** | **Ash (g)** | **Calcium**  **(g)** | **Phosphorus (g)** | **Ca:P ratio** | **Potassium (g)** | **Magnesium (g)** | **Sodium**  **(g)** | **Copper (mg)** | **Iron (mg)** | **Manganese (mg)** | **Zinc (mg)** |
| --- | --- | --- | --- | --- | --- | --- | --- | --- | --- | --- | --- | --- | --- | --- | --- | --- |
| NRC | - | - | 50.00 | 22.50 | - | - | 0.72 | 0.64 | - | 1.30 | 0.10 | 0.17 | 1.20 | 20.00 | 1.20 | 18.50 |
| FEDIAF | - | - | 83.30 |  | - | - | 1.97 | 1.67 | 1(min)/2(max) | 2.00 | 0.13 | 0.25 | 0.1.67 | 26.70 | 1.67 | 25.00 |
| Diet 24 | 13.81 | 3.60 | 25.64 | 3.72 | 17.43 | 14.23 | 0.57 | 1.00 | 0.57 | 1.28 | 0.41 | 0.15 | 2.13 | 12.61 | 10.07 | 8.04 |
| Diet 25 | 25.47 | 4.39 | 182.58 | 29.59 | 3.30 | 7.58 | 0.24 | 1.61 | 0.15 | 0.85 | 0.22 | 0.30 | 15.13 | 12.15 | 0.02 | 3.54 |

Ca. calcium. P. phosphorus.
